# Which experimental procedures influence the apparent proximal femoral stiffness? A parametric study

**DOI:** 10.1186/s12891-021-04656-0

**Published:** 2021-09-23

**Authors:** Morteza Amini, Andreas Reisinger, Lena Hirtler, Dieter Pahr

**Affiliations:** 1 Institute of Lightweight Design and Structural Biomechanics, TU Wien, Getreidemarkt 9, Vienna, 1060 Austria; 2 Division Biomechanics, Karl Landsteiner University of Health Sciences, Dr.-Karl-Dorrek-Straße 30, Krems an der Donau, 3500 Austria; 3 Center for Anatomy and Cell Biology, Medical University of Vienna, Währinger Straße 13, Vienna, 1090 Austria

**Keywords:** Biomechanics, Femur, Mechanical properties, Parametric study, Apparent stiffness

## Abstract

**Background:**

Experimental validation is the gold standard for the development of FE predictive models of bone. Employing multiple loading directions could improve this process. To capture the correct directional response of a sample, the effect of all influential parameters should be systematically considered. This study aims to determine the impact of common experimental parameters on the proximal femur’s apparent stiffness.

**Methods:**

To that end, a parametric approach was taken to study the effects of: repetition, pre-loading, re-adjustment, re-fixation, storage, and *μ*CT scanning as random sources of uncertainties, and loading direction as the controlled source of variation in both stand and side-fall configurations. Ten fresh-frozen proximal femoral specimens were prepared and tested with a novel setup in three consecutive sets of experiments. The neutral state and 15-degree abduction and adduction angles in both stance and fall configurations were tested for all samples and parameters. The apparent stiffness of the samples was measured using load-displacement data from the testing machine and validated against marker displacement data tracked by DIC cameras.

**Results:**

Among the sources of uncertainties, only the storage cycle affected the proximal femoral apparent stiffness significantly. The random effects of setup manipulation and intermittent *μ*CT scanning were negligible. The 15^∘^ deviation in loading direction had a significant effect comparable in size to that of switching the loading configuration from neutral stance to neutral side-fall.

**Conclusion:**

According to these results, comparisons between the stiffness of the samples under various loading scenarios can be made if there are no storage intervals between the different load cases on the same samples. These outcomes could be used as guidance in defining a highly repeatable and multi-directional experimental validation study protocol.

## Introduction

Validation of numerical predictive and monitoring models is carried out against experimental results. This validation process determines how accurate a model can mimic reality [1, 2]. Image-based methods, such as CT-based finite element (FE) models, have become state-of-the-art in biomechanical bone research, with clinical use cases [3–7]. Using these models, the risk of fracture in patients with underlying conditions, such as osteoporosis, can be non-invasively estimated to guide treatment efforts and lessen the consequent immobilization burden.

The most prevalent site in orthopedic biomechanics studies is the hip or proximal femur. Hip fractures account for the majority of fracture-related disabilities [8, 9]. Osteoporosis and fall are the two leading causes of hip fractures [10, 11]. Many biomechanical experiments done on femur are based on single tests per sample [12–18]. To measure the strength of the bone, samples are loaded until failure. FE models validated against such experimental data are at risk of being biased towards those specific experimental load cases.

There has been growing evidence that considering multiple loading directions in the experimental and numerical studies might improve the predictive ability of FE models [5, 19–21]. In one study [19], using nonlinear CT-FE models, the effect of loading direction on the fracture load and location of the proximal femur was investigated. However, the employed FE model was only validated against a single stance load case [22]. In other studies [23, 24], samples were loaded in multiple fall load cases to determine the accuracy of the FE-predicted strains related to the side-way fall incidents. Although one FE technique [23] was previously validated against quasi-axial load cases as well [2, 7, 25], the two studies were done on different sample groups, one for stance and one for the fall. To our knowledge, there are no studies with multiple loading directions applied in both stance and fall configurations on the same samples.

In order to perform multiple mechanical tests on each sample, structural damages should be avoided through non-destructive loading regimes. This imposes two restrictions: First, the load amplitude should be restricted to much lower values compared to fracture loads. Second, a surrogate measure for the sample strength should be employed. A widely used criterion to address the former is conducting non-destructive tests on the femur by applying a fraction (75%) of the donor’s body weight as the maximum load [26]. With regards to the latter, the apparent stiffness, a frequently reported surrogate bone strength measure, can be used as the outcome variable [27]. Despite the above-mentioned restrictive circumstances, using elastic structural metrics (i.e., apparent stiffness) might still seem like a step backward. However, if the apparent stiffness shows significant alterations, it will point to the direction of a change in the tested material itself, which in turn would affect the strength [28, 29].

To compare the apparent stiffness of the samples under different loading conditions, other unwanted experimental sources of alteration in sample stiffness must be determined. These uncertainties often arise from simplifications introduced during the experimental procedures and can be investigated using a parametric approach. This study design is a powerful tool to isolate the influence of all players and compare their relative significance systematically [30, 31]. Most studies have only reported the repeatability measures of the experiments via repeating each test case multiple times [2, 32] or by re-orienting and re-installing the setup between each test [26]. Others have studied the effect of FE modeling methodological determinants on predicted femoral strength [3]. To our knowledge, there are no studies investigating the effect of typical experimental parameters on the structural properties of the samples using a systematic approach.

The aim of this study was to parametrically determine the influence of common steps involved in an experimental validation study on the apparent stiffness of the proximal femur under multiple loading directions, namely: repetition, pre-loading, re-adjustment, re-fixation, storage, *μ*CT scanning, and loading direction (15^∘^ deviation from neutral alignment) in stance and side-fall configurations. To our knowledge, this is the first study in which both stance and fall configurations have been tested on every sample. These results could add an aggregated reference to the currently scattered pool of data required for planning experimental protocols with reduced uncertainties affecting the measured structural properties of bone samples.

## Methods & materials

### Samples

Ten proximal femoral samples from five donors (Table 1) were harvested and kept frozen in −23^∘^ (Center for Anatomy and Cell Biology, Medical University of Vienna). The specimens originated from voluntary body donations for scientific and teaching purposes to the Center (According to protocol accepted by the ethics committee of Karl Landsteiner University of Health Sciences). Samples were screened for lack of any pathological disease. All procedures were performed in accordance with relevant guidelines.

**Table 1:** Information on donors of the samples

Donor ID	Sample ID	Gender	Age	Height (m)	Weight (kg)
**#1**	9119-9120	F	63	1.53	65.4
**#2**	9390-9391	F	69	1.67	49.3
**#3**	9628-9629	F	70	1.67	48.1
**#4**	9270-9271	M	78	1.71	72.0
**#5**	9247-9248	M	78	1.77	66.8

Both femora from each donor were tested (10 samples). Only samples with no pathological conditions were included in the study

### Experimental setup

To perform a parametric study, we developed a new femoral experimental setup based on two main criteria: 1. possess fully defined boundary conditions, and 2. provide the means for fast multiple non-destructive tests in stance and fall configurations with variable loading direction. The test setup was comprised of the following main components: Alignment setup, embedding components, testing apparatus, scan chambers, and DIC setup.

**Fig. 1:**
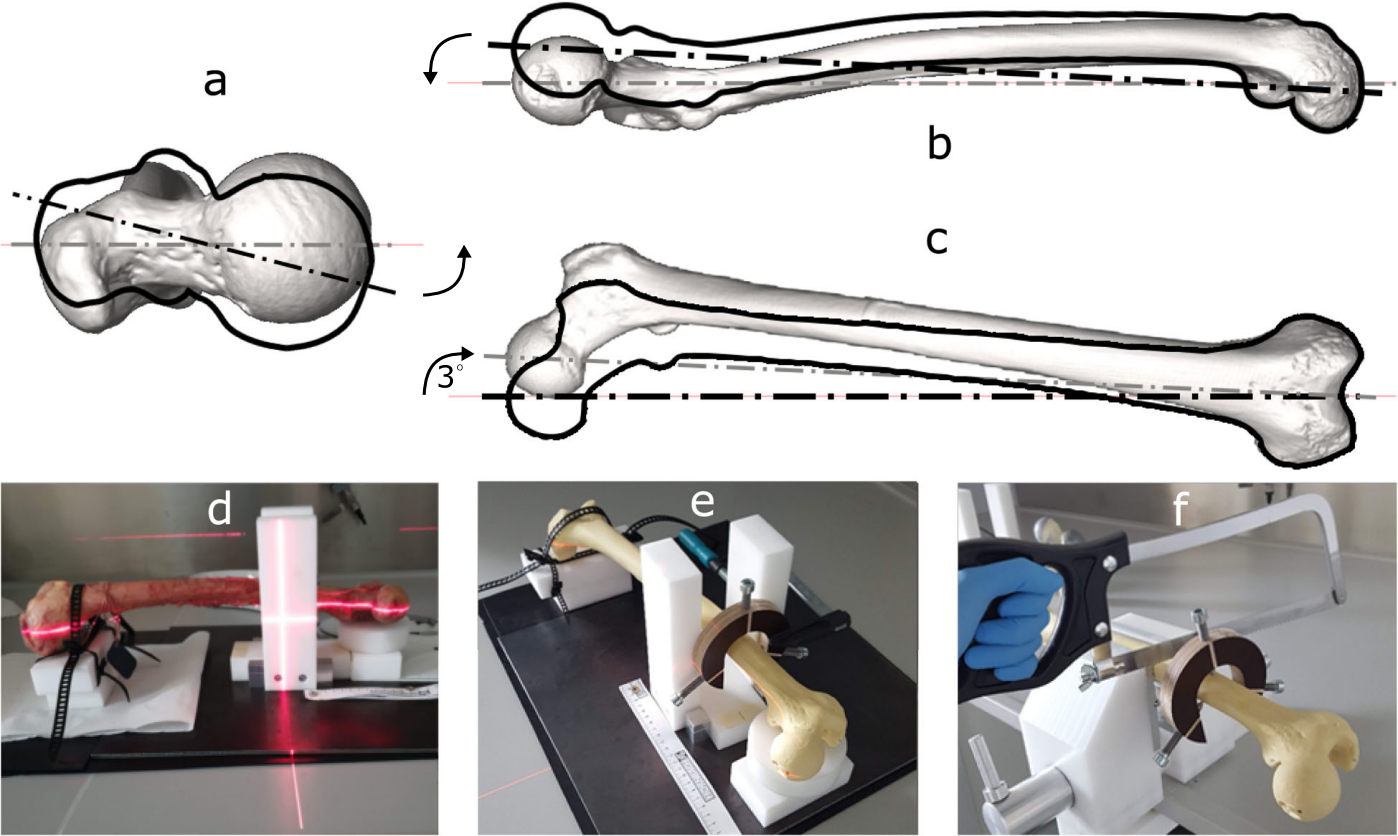
The neutral stance alignment of the femur was achieved by using a custom-made alignment setup. The intact femur was a) tilted around the shaft axis until the neck axis was horizontal, b) leveled so that the femoral head center and the distal mid-condylar line were coincident, and c) tilted in the adduction direction until its mechanical axis was 3^∘^ beyond the reference line. These steps were achieved using d) two cross lasers, and e) manufactured POM supports. The aligned sample was f) cut to a sample size of 150 mm, measured from the femoral head along the shaft axis

**Alignment setup:** A custom-made alignment setup was used to maintain the femur’s physiological neutral stance alignment in the final prepared sample. It was comprised of two cross lasers, in-house 3D printed holder devices, and manufactured POM (Polyoxymethylene) supports (Fig. 1). The intact femur was laid down on two distal and proximal supports. The bone was axially tilted until the neck axis, passing through the femoral head center and middle of the femoral neck, was coincident with the horizontal line of a cross laser (Fig. 1a). Metal spacers with various thicknesses were placed between the condyles of the femur and the distal support to maintain the torsional alignment. Then, the proximal support was elevated using a screw mechanism so that the femoral head center and the distal mid-condylar line were coincident with the horizontal laser line (Fig. 1b). Finally, using the second cross laser mounted above the dissection table, a 3^∘^ adduction angle with the reference line of the alignment supports was formed. The femoral head center and the femoral intercondylar fossa, hence the mechanical axis, were aligned with the laser line (Fig. 1c). Once all angles were determined, a custom-made C-shaped device was used to fix the alignment on the proximal portion of the bone making it ready for the cutting and potting steps. This device was fixed 105 mm below the bounding plane coincident to the most proximal point of the femoral head (Fig. 1e). The proximal portion of the bone was cut using a bone saw 45 mm below the fixed device, resulting in a total sample length of 150 mm (Fig. 1f). The total sample length was restricted by the maximum field of view from the *μ*CT scanner, which was necessary for future studies using the micro-FE models of the samples.

**Fig. 2:**
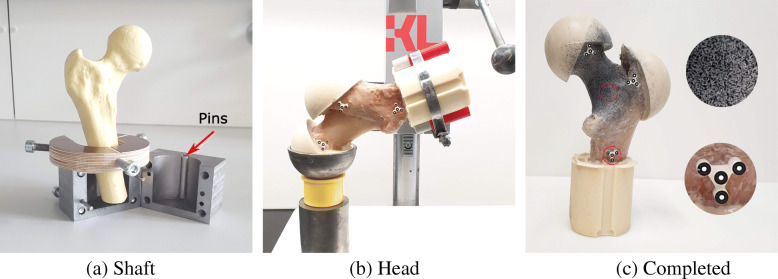
The proximal portion of samples was potted at (a) shaft, and (b) head and trochanter locations using Polyurethane (PU) casting resin and a custom-made setup. (c) The posterior femoral neck area was sprayed with an airbrush to create a speckle pattern (trial tests). Three marker clusters containing four markers each were placed on the femoral head, trochanter, and shaft of the prepared samples

**Embedding components:** The proximal femoral samples were held in their neutral stance alignment on the potting block, and their shaft was embedded in a 50 mm diameter cylinder. A 5-mm gap was left between the bottom of the sample and block to account for uneven cutting surfaces. Two pins were attached to the walls of the potting block to prevent the shaft from rotating in the holder (Fig. 2a). The trochanter and the head were embedded in spherical segments to provide defined boundary conditions at the contact points and avoid local crushing. The alignment was done using 3D printed adapters (Fig. 2). Using printed holders and pins, 4-marker clusters were later attached to the femoral head, major trochanter, and shaft of the samples for displacement tracking.

**Fig. 3:**
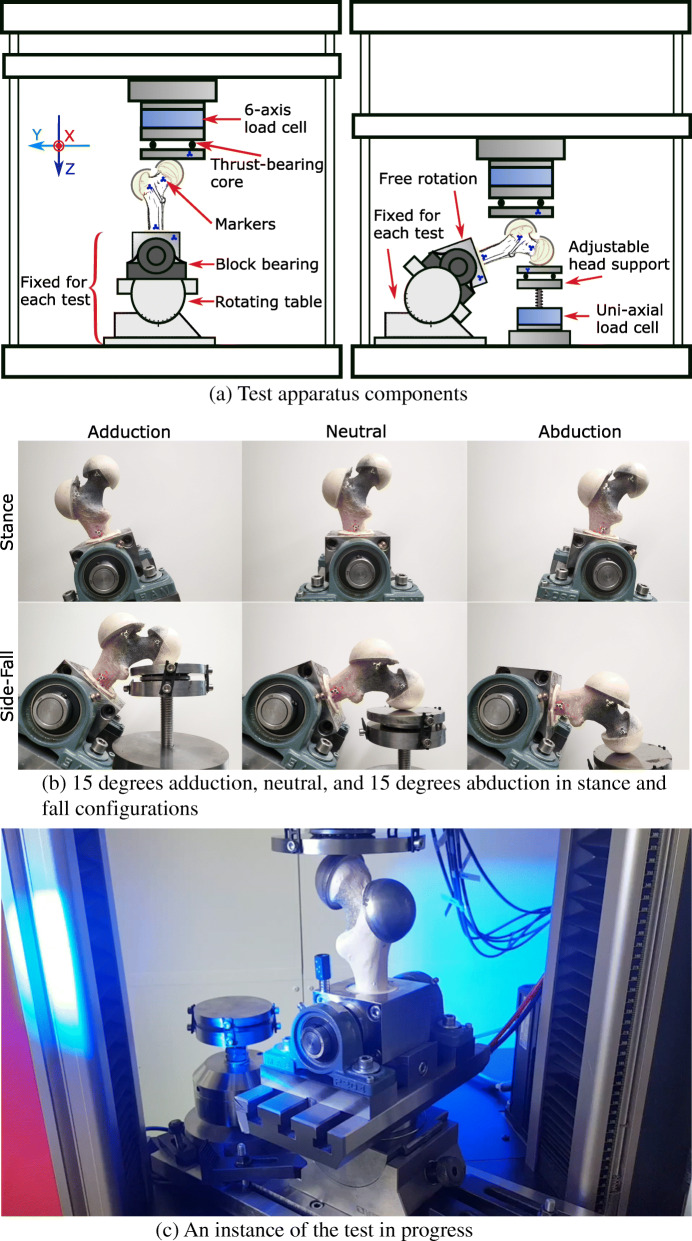
The testing apparatus allowed for multiple loading directions in both stance and fall configurations

**Testing apparatus:** A 25 kN load cell with 6 degrees of freedom (DOF) (Hottinger Baldwin Messtechnik (HBM) GmbH, Germany) was mounted on the 30 kN electro-mechanical axial testing machine (Z030, ZwickRoell Ulm, Germany). Hardened iron disks were manufactured and used with ring ball-bearings to apply a purely axial load (Fig. 3). A rotating milling machine table was equipped with a hinge bearing to hold the shaft of the sample and allow for 5 degrees of freedom for the sample alignment (X, Y, Rx, Ry, Rz). An additional uni-axial 25 kN load cell (see Fig. 3) was used in the fall configuration to support the head while the trochanter is loaded (HBM, Germany). In the stance configuration, the shaft block was fixed on the rotating table. The abduction\adduction angle was adjusted on the table. The table was fixed on the testing machine. In the fall configuration, the shaft block was free to rotate in the abduction\adduction direction while the head was resting on the support load cell. The table was fixed on the machine as well (Fig. 3).

**Fig. 4:**
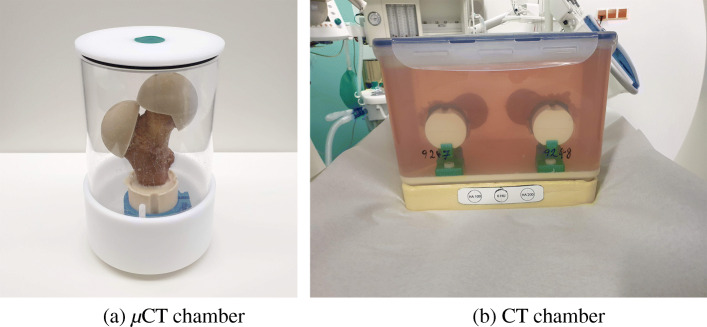
a) *μ*CT chamber from POM and Plexiglass to fix the sample in neutral alignment and keep it hydrated throughout the scanning process, b) CT chamber from polypropylene plastic fitted with embedding base, and holders to accommodate a pair of samples submerged in saline solution and in neutral alignment

**Scan chambers:***μ*CT: A custom-made chamber was manufactured using POM (Polyoxymethylene) and Plexiglas (Fig. 4). The cylindrical chamber was 15 cm in diameter and 17 cm inner height. A clamping mechanism was fitted in the chamber so that the femur could be stood upright and fixed to avoid movement artifacts during the scan. A sealing cap with a pressure valve was used to make sure the sample is not dehydrated during the scan while the heated air can escape the chamber.

CT: To mimic the clinical conditions in which two legs with surrounding soft tissues are present in scanner’s field of view, we modified a rectangular translucent storage box (polypropylene) using embedding material, 3D printed adapters, and ready-made PVC holders. Each pair of samples were fixed side-by-side, in their neutral alignment, 20 cm apart, and fully submerged in saline solution (Fig. 4).

**Fig. 5:**
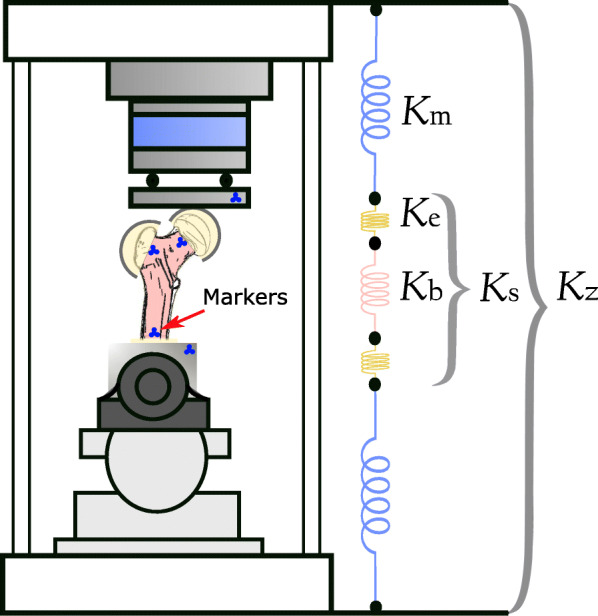
Stiffness of the machine (*K*_m_) and the embedding (*K*_e_) could be calculated based on the stiffness of the whole assembly (*K*_z_), the bone (*K*_b_), and full specimen (*K*_s_) measured using markers (depicted in blue). These values were used to verify the methodology and the outcome variable

**DIC setup:** Digital Image Correlation (DIC) system (ARAMIS 3D Camera, GOM GmbH, Braunschweig, Germany) with two CCD cameras was used for optical displacement tracking. The 6-megapixel cameras were 150 mm apart and positioned at a perpendicular distance of 350 mm from the sample, capturing images at a 10 Hz rate from a measurement volume of 160 x 130 x 95 mm (LxWxD). All measurements were done according to the manufacturer’s standard protocol (GOM GmbH, Braunschweig, Germany). The system was calibrated before beginning of each session using the standard calibration plate and according to the manufacturer’s protocol keeping the calibration deviation below 0.05 pixels. Clusters of markers comprised of four markers (GOM GmbH, Braunschweig, Germany) attached to a 3D printed holder were placed on the head, trochanter, and shaft of the samples in order to measure the apparent stiffness of the bone (Fig. 2). Additional markers were placed on the loading plate and holder block in order to be able to measure the apparent stiffness of the full specimen as well (Fig. 5). All markers were covered within the measurement volume at all time. There was a 1 micron displacement noise in the marker displacement data at zero load.

### Parameters

While all steps involved in our experimental validation study are described below, not all the acquired data were relevant and hence presented in this manuscript’s scope (e.g., scan data acquired for the FE modeling phase elsewhere). The following parameters were tested for stance and side-fall configurations:

- *Repetition:* Repeating a test five times without touching any parts of the setup or sample.
- *Pre-loading:* Testing a load case once right after fixing the sample into the desired place and alignment and comparing the results with the average of the immediately following five repetitions, without touching the sample or setup in between.
- *Re-adjustment:* Distorting the sample configuration and placement on the machine and re-adjusting it back to the initial condition without taking the sample out of the setup.
- *Re-fixation:* Taking out the sample and distorting the setup adjustments, then putting everything back to their initial condition.
- *Storage:* Storing samples in a −23^∘^C freezer for four weeks.
- *μCT scanning:μ*CT scanning the sample.
- *Loading direction:* Tilting the samples for ± 15 degrees along the abduction-adduction axis from their neutral stance or fall alignments.

### Study design

Three sets of biomechanical testing were carried out according to the following plan (Fig. 6). For each test, samples were loaded up to 75% of the donor’s body weight (BW) to avoid any damage or destruction [26]. Loading was applied at a 5 mm/sec rate. There was a minimum of 1-minute pause between each consecutive tests (3 or 6 minutes for tests requiring switching between the direction or configuration of the sample, respectively):

1. Fresh frozen samples were taken from the freezer one-by-one, and the excess muscle and fat tissue was cut from their proximal half using a knife and scalpel. The periosteum was carefully scraped from the femoral neck region as well as the shaft using bone scraper. The greater trochanter surface was scraped to remove cartilaginous tissues, but the femoral head cartilage was kept intact to avoid damaging the thin cortex at that region. The clean sample was then aligned, cut, and embedded in a span of 3 hours, and stored back in the freezer until all samples were processed. The process was done on frozen samples since the soft tissue removal was easier than the thawed bone, and the bone marrow could be sealed inside the bone, avoiding large air cavities in the bone for better scan qualities.

**Fig. 6:**
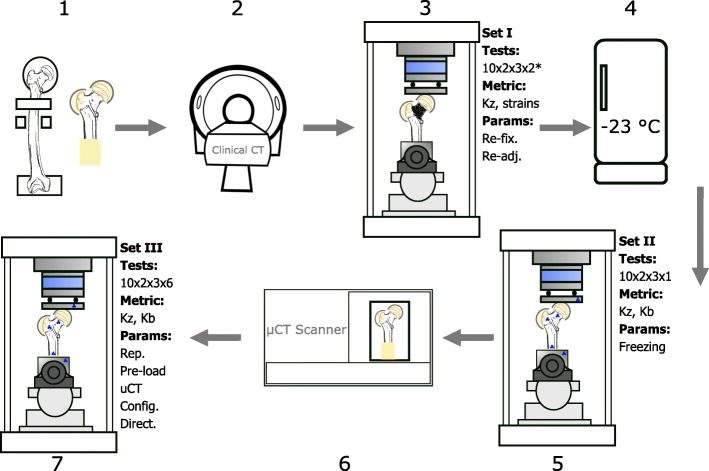
1. Fresh frozen specimens aligned, cut, and potted in neutral stance position, 2. Clinical CT scan of the samples (data not presented, acquired for another study), 3. The first set of mechanical tests (set I): 10 samples x 2 configurations x 3 directions x 2 tests per load case (* a third test per load case was done on one sample for re-fixation), collecting: apparent stiffness and surface strain data, evaluating: re-fixation and re-adjustment, 4. Samples stored at −23^∘^C for four weeks, 5. The second set of tests (set II): 10 samples x 2 configurations x 3 directions x 1 test per load case, collecting: apparent stiffness and marker data, evaluating: storage, 6. *μ*CT scan of the full sample (5 hours per scan), 7. The third set of tests (set III): 10 samples x 2 configurations x 3 directions x 6 repeated tests per load case, collecting: apparent stiffness and marker data, evaluating: repetition, pre-loading, *μ*CT scanning, and loading configuration and direction
2. On the day of the CT scanning, all samples were submerged in 0.9% PBS (Phosphate-buffered saline) solution filled plastic bags and placed in the vacuum-desiccator (Trivac D8B; OC Oerlikon Management AG, Pfäffikon, Switzerland) for 30 minutes at room temperature to thaw and extract the air bubbles. The samples were then carefully transferred in their submerged condition into their corresponding room-temperature PBS-filled CT chambers and fixed. After scanning (Toshiba Aquilion Prime, res: 0.625x0.625x0.25 *mm*^3^), samples were wrapped in soaked towels and stored at −23^∘^C for one week. CT data is necessary for the CT-based FE modeling phase following this study. Given the relatively short scan time (13 secs compared to 5 hrs for *μ*CT) and ideal submerged sample conditions throughout the process, it was not included as a parameter in the study (to reduce the number of test rounds).
3. Set I: Frozen samples were thawed in a room temperature PBS solution bath for 4 hours. The posterior femoral neck area was pat dried and degreased using ethanol pads. Speckle pattern was sprayed. Each sample was tested in stance and side-fall configurations for each of the three loading directions (neutral, 15^∘^ abduction, 15^∘^ adduction). These six load cases were repeated twice to examine the effect of re-adjustment for all samples. Additionally, for one sample all 6 load cases were repeated once more to check the re-fixation effect (due to the time consuming nature of removing the sample from the setup for every test, this parameter was limited to only 1 sample).
4. Samples were wrapped in soaked towels and stored for four weeks in a −23^∘^C freezer.
5. Set II: Frozen samples were fully thawed in room temperature PBS bath. Marker clusters were pinned on the head, trochanter, and shaft of the sample on the posterior side. Each sample was tested in stance and side-fall configurations for each of the three loading directions. Tests were done only once. Markers were tracked using stereo cameras.
6. *μ*CT scanning: Samples were taken out of the testing setup, submerged in PBS solution bath for re-hydration, clamped in the scan chamber, with soaked towels placed at the bottom of the sealed (with a pressure valve) chamber to avoid dehydration. The full sample length was scanned in a 5-hour session (Skyscan 1173, Bruker, Belgium) (field of view: 120 x 150 mm, resolution: 30 *μ*m, voltage: 130 kV, current: 60 mA, exposure: 580 ms, filter: Al 1.0 mm).
7. Set III: It was done immediately after the *μ*CT scanning and on the same day as set II. Each sample was tested in stance and side-fall configurations for all three loading directions. Each test was repeated 6 times (with a one-minute resting period in between) to examine the repeatability of the tests as well as the pre-loading effect. Samples were frozen at the end.

A total of 180 tests (and an additional 66 and 300 repetitions in set I and set III, respectively) were performed. Data was captured at 100 Hz and 10 Hz by the testing machine and stereo cameras, respectively.

### Data analysis

Collected raw data was comprised of: axial and shear loads as well as moments at the load introduction site, femoral head support force in side-fall load cases, vertical displacement of the machine head, and marker displacements at the five locations of : loading plate, femoral head, major trochanter, shaft, holder block. The support load, shear loads, and moments were used to check the boundary conditions of the setup. Analytically, we should expect zero shear forces at the load introduction location to match the free horizontal translation DoFs. Furthermore, the maximum moment at the loading plate should match the values calculated using the maximum load and the distance between the center of contact surface between the femoral head/trochanter cap (in stance and side-fall configurations, respectively), and the center of the load cell. A significant difference between the experimental and analytic results would point into direction of unwanted bending moment on the femoral head, negating free rotational DoFs. Finally, the reaction force at the support plate in side-fall configuration is calculated using the distances between the loading and support contact points and the shaft bearing axis. Significant deviation from this value would contradict with free rotation DoF at the shaft (Fig. 3a)

**Fig. 7:**
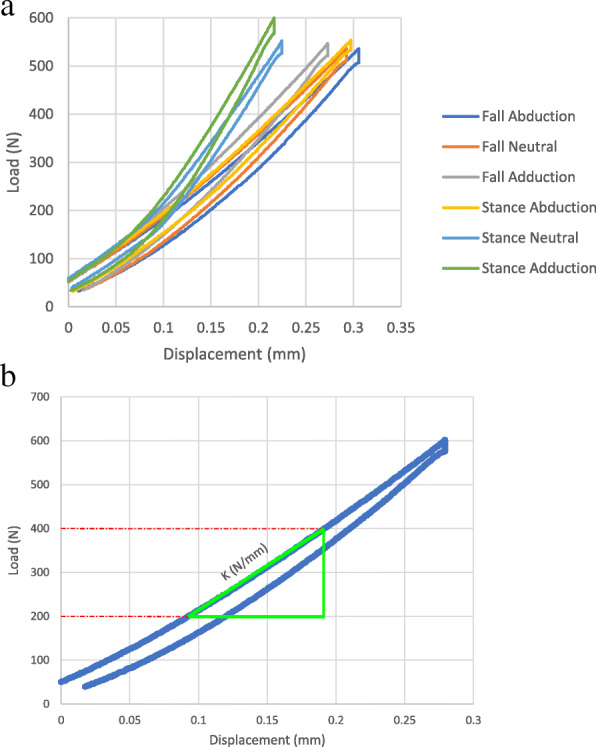
a) Load-displacement plots from the testing machine showing the full loading and unloading cycles for all six load cases of a sample. b) The overall stiffness of the sample for each test was defined as the slope of the linear section of the plot, which was set between 200 N and 400 N for all samples
- *Bone stiffness*(*K*_b_): the relative vertical displacement of the femoral head and shaft (for stance) or trochanter and femoral head (for fall) markers.

**Outcome variables:** The apparent stiffness was defined as the slope of the linear section of the load-displacement curve (Fig. 7). Based on some preliminary tests, the linear section was defined between 200N and 400N. The load came from the axial component recorded by the 6 DOF load cell. Depending on the stiffness measurement criteria, displacement was based on (Fig. 5):

- *Sample stiffness*(*K*_z_): the moving head of the mechanical testing machine.

The stiffness of the embedding segments and the machine components (*K*_e_ and *K*_m_, respectively) were calculated using the spring theory.

**Fig. 8:**
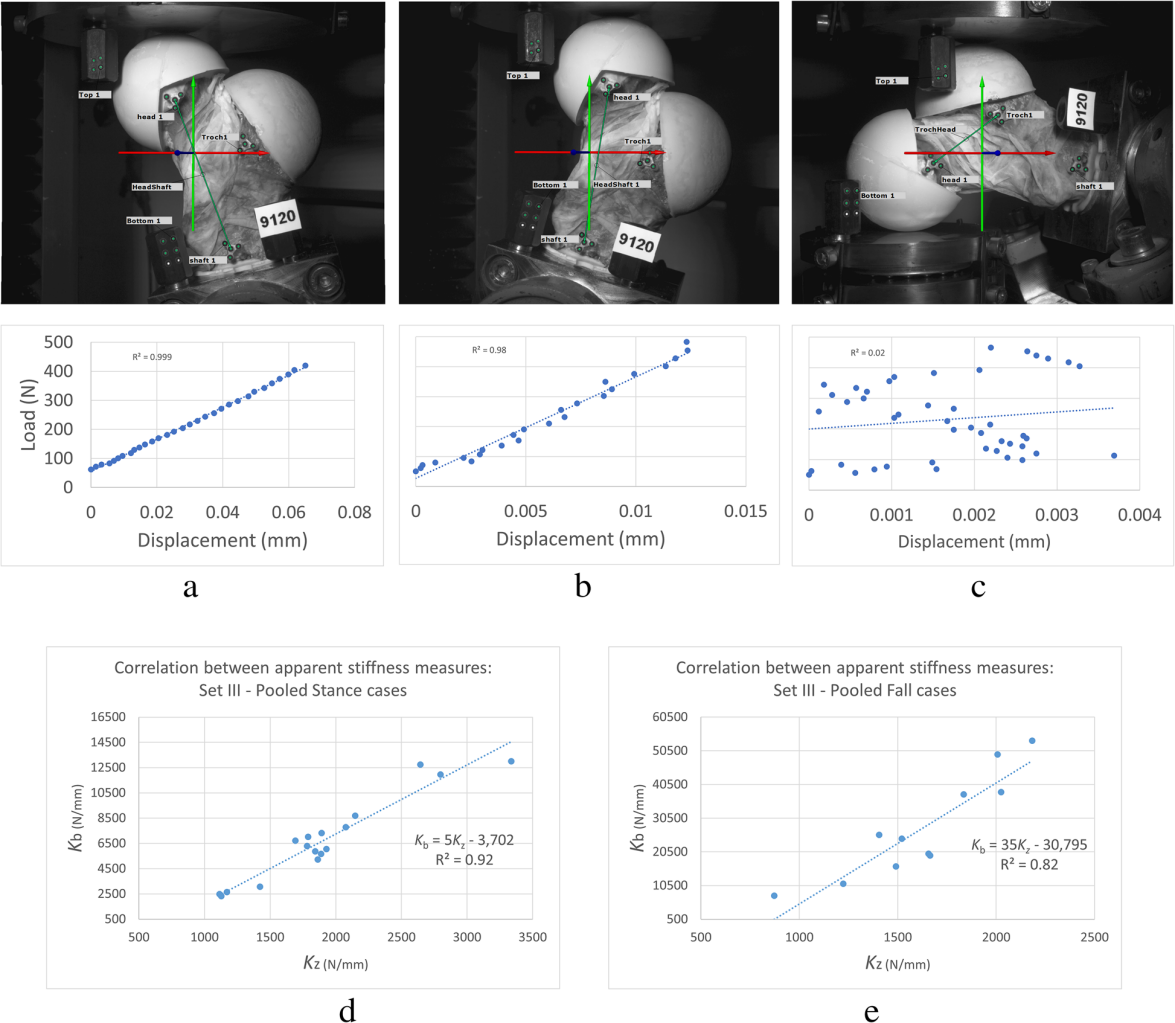
The 75% BW peak load resulted in relatively small displacements in the bone. Depending on the configuration, direction, and stiffness of the sample, the recorded values for marker displacements had different qualities. The example plots show instances with *R*^2^ values of the regression line of (a) 1.00 (0.9997), (b) 0.98, and (c) 0.02 corresponding to stance abduction, stance adduction, and fall abduction load cases of one sample, respectively. Applying a *R*^2^>0.95 threshold for the marker load-displacement data to be considered viable, apparent stiffness measures of *K*_b_ and *K*_z_ showed good correlation in (d) stance (*R*^2^ = 0.92) and (e) fall (*R*^2^ = 0.82) configurations

Markers were tracked by the DIC cameras and load-displacement plots were evaluated. Tests with highly linear load-displacement plots (*R*^2^>0.95) were considered for further analysis. *K*_z_ was validated against the corresponding *K*_b_ data and used as the main outcome variable (Fig. 8).

**Statistical Analysis:** To report the tests’ repeatability, we used the coefficient of variation or CV% by dividing the standard deviation by the average of the five repeated measurements of each load case in set 3:

$$CV\% = \frac{Standard\:Deviation}{Average} $$

Based on the normality test on the data sets, either the Wilcoxon t-test or Student’s t-test were used to determine the significance of the difference between paired groups, with a significance threshold set at 0.05. To ensure the validity of the t-tests, we imposed a minimum requirement of 6 data points per parameter. Where data from set III was involved, the average of the five repetitions was considered for the analysis. For the re-fixation parameter, where only one case was tested multiple times, a percent difference (%Diff) between the average of the five repetitions before and after re-fixing the sample was reported.

## Results

**Boundary conditions:** No substantial peak shear forces (< 2*N*) at the load introduction site were recorded across all tests. The calculated and measured support reaction forces for the fall configuration were matching. These findings validated the defined boundary conditions of the setup.

**Repeatability parameters:** The average repetition error (CV%) in *K*_z_ measurements for all load cases was 1.53% (95% confidence interval [0.32, 2.75]). Re-fixation, Pre-loading, and Re-adjustment did not affect the *K*_z_ significantly (Table 2).

**Table 2:** Effect of tested parameters on the apparent stiffness (*K*_z_) of the proximal femoral samples

Parameter	Avg. %Diff	SigMethod	P	EffectSize
**Re-fixation**	-3.74	NA	NA	NA
**Pre-loading**	0.70	Wilx.T	0.45	NA
**Re-adjustment**	-5.35	Std.T	0.38	0.15
**Storage**	25.46	Wilx.T	HTML 0061000.00	NA
***μ*CT scanning**	-3.92	Std.T	0.92	0.02
**Stance-Adduction**	10.92	Std.T	0.11	0.55
**Stance-Abduction**	-33.22	Std.T	HTML 0061000.00	2.54
**Fall-Adduction**	-4.90	Wilx.T	0.17	NA
**Fall-Abduction**	12.91	Wilx.T	HTML 0061000.02	NA

The first half of the table pertains to the unwanted sources of uncertainties, while the second half contains the controlled alteration of the test conditions. Based on the normality test results for the samples, either Student’s or Wilcoxon T-test was used for the significance test. Significant results are highlighted by green background. For the re-fixation parameter, due to lack of sufficient sample size, only the average % Diff is reported. The average repetition error (CV%) for all load cases was 1.53% (95% confidence interval [0.32, 2.75])

**Sample manipulation effects:** The storage cycle significantly reduced the *K*_z_ of the samples (*p*-value < 0.01, avg. % Diff ≈ 25%). The *K*_z_ showed no significant effect from performing *μ*CT scanning on the samples (p-value =0.92).

**Fig. 9:**
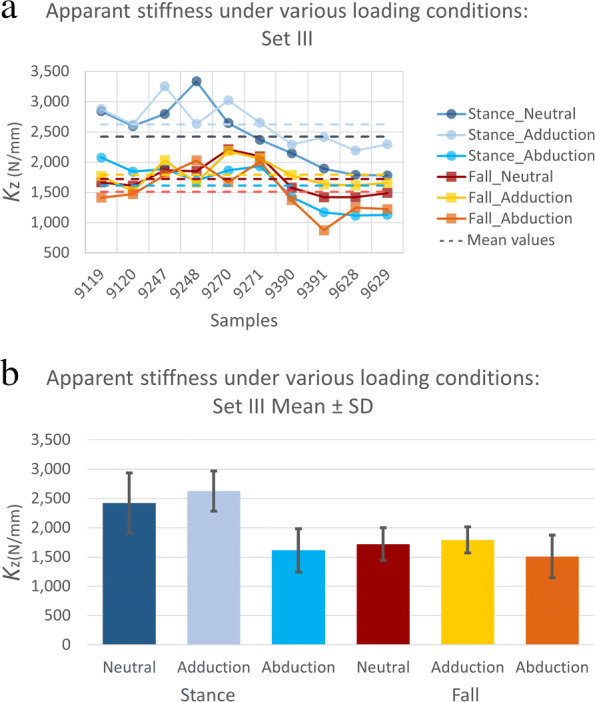
(a) Apparent stiffness of the samples (*K*_z_) under different load conditions. Stiffness values are the average of 6 repeated measurements per load case in set III. Horizontal dashed lines represent the average value of the load case with corresponding color. (b) The average stiffness of the 10 samples per loading condition with error bars representing one standard deviation (SD) and following the same color code

**Test configurations:** The measured *K*_z_ for the neutral stance alignment was 27% larger compared to the neutral fall (p < 0.01). The samples were, on average, 33% and 13% less stiff when abducted for 15 degrees in the stance and fall cases, respectively (p < 0.02) (Table 2). Deviation of the load direction by 15 degrees in the adduction direction did not alter the apparent stiffness significantly. The overall trend of stiffness values for each load configuration can be seen in Fig. 9.

**Fig. 10:**
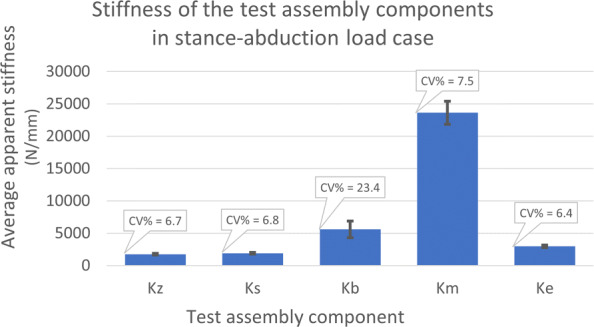
The average apparent stiffness of test assembly constituents in the stance-abduction load case. The only variable element across the tests were the samples. Components are: Full testing assembly (*K*_z_), full specimen comprised of the bone and embedding caps (*K*_s_), the bone segment between the femoral head and shaft markers (*K*_b_), testing machine including the tilting table and load cells (*K*_m_), and embedding material (*K*_e_)

**Stiffness measurement:** To validate the *K*_z_ values, which are based on the machine displacement data, we plotted load-displacement graphs using marker tracking data (Fig. 8). Among all load cases, stance-abduction configuration produced highly linear results (*R*^2^>0.95) for all ten samples followed by stance-neutral with 7 viable data points. There were a total of 11 fall configuration tests falling into this criteria as well. There was a strong correlation with an *R*^2^ ≈ 0.92 and 0.82 between the machine and marker data for stance and fall, respectively (Fig. 8(d,e)). Furthermore, the sample stiffness, *K*_z_, was decomposed into its constituent elements using the spring theory (Fig. 5). The bone and full specimen values, *K*_b_ and *K*_s_, were measured using marker tracking. The machine and embedding stiffness values, *K*_m_ and *K*_e_, were calculated accordingly (Fig. 10):

$$\begin{array}{*{20}l} \frac{1}{K_{\text{e}}} = \frac{1}{K_{\text{s}}} - \frac{1}{K_{\text{b}}}\\ \frac{1}{K_{\text{m}}} = \frac{1}{K_{\text{z}}} - \frac{1}{K_{\text{s}}} \end{array} $$

Looking at the percent coefficients of variation (CV%), the variation in *K*_z_ appears to be mostly stemming from the relatively high variability of *K*_b_, with the mechanical components showing fairly consistent stiffness measurements. In the given load case, the *K*_z_ was three times softer than the *K*_b_, influenced largely by the *K*_e_.

## Discussion

In this study, we aimed at using a systematic approach to find which experimental parameters affect the apparent stiffness of the proximal femur. Our results indicate that among the sources of uncertainties, the storage of the samples significantly alters their apparent stiffness. Moreover, controlled parameters, i.e., loading direction, also has significant effects on the apparent stiffness of the proximal femoral samples. Other sources of random effects pertaining to the repeatability of the mechanical tests proved negligible.

**Fig. 11:**
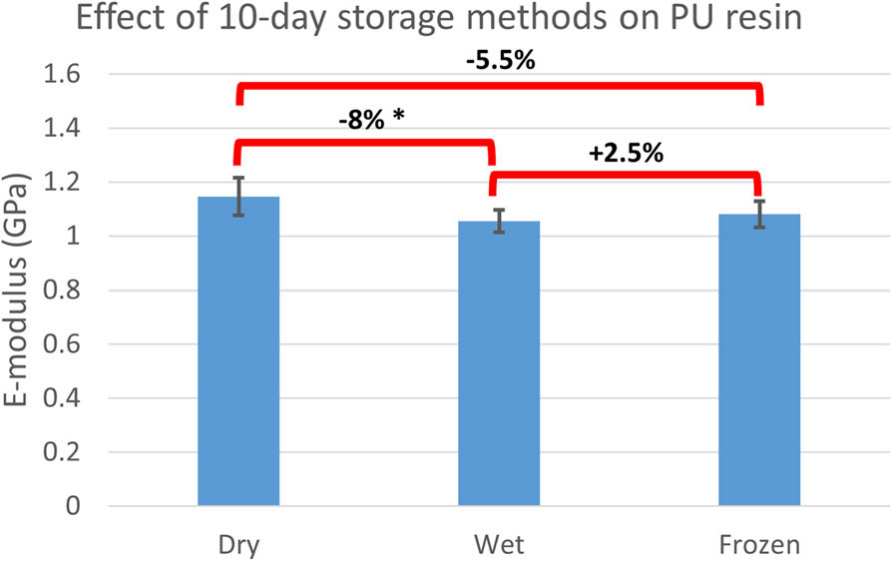
Effect of storage methods on the Polyurethane (PU) resin tensile E-modulus. Ten standard (DIN ISO 527-2) tensile samples per storage method was produced. They were stored either in a dry container (dry), submerged in a 0.9% PBS solution bath (wet), or wrapped in soaked towels and sealed in a −23^∘^C freezer (frozen) for ten days. The average tensile E-modulus of each group was acquired through standard tensile tests. Student’s T test was used for comparison between the means (significance at P 0.05)

According to our findings, a freezing and storage cycle would significantly affect the apparent stiffness (p < 0.05). A 25% alteration in the *K*_z_ was measured. A cycle included storing wrapped and sealed samples in a −23^∘^C freezer for four weeks. This could potentially mean that comparisons between stiffness measurements before and after a storage cycle might be critically compromised and should be ideally avoided in experimental validation studies. Our results are different from previous reports on the subject. Some earlier research on the effect of storage methods on trabecular bone [33] or skull [34] reported no change in the stiffness of their specimens after several freezing and storage cycles. The inconsistencies between these outcomes might have roots in the sample choices. We have tested full proximal femur samples, meaning the embedding caps and cartilage layers were also frozen and tested attached to the bone, while the aforementioned studies used bare bone samples. Among possible pathways for the observed structural behavior, incomplete thawing or dehydration of the samples during the tests could be readily rejected given the insignificant difference between set II and set III and the samples’ hydrated conditions throughout storage and tests. Another hypothesis is that the freezing cycle might affect the embedding-cartilage or cartilage-bone conjunctions rather than the bone structure. To check the effect of similar storage cycles on the embedding material, three groups of 10 standard tensile test specimens (DIN ISO 527-2 b) were produced using a 3D printed cast. They were stored a) in a dry container, b) submerged in 0.9% PBS bath, and c) wrapped in soaked paper towels and sealed in the −23^∘^C freezer for 10 days. Afterwards, frozen samples were thawed in room temperature PBS bath and all samples were tested to measure their tensile E-modulus (DIN ISO 527-2). Student’s T test was used for comparing the groups. There was no significant difference between the dry-frozen and wet-frozen groups. There was an 8% decrease in E-modulus of the wet samples compared to the dry group (Fig. 11). Since according to the average stiffness values in Fig. 10, a 40% increase in *K*_e_ is required to account for the observed 25% increase in the *K*_z_ post storage cycle, the embedding material seems an unlikely candidate. Based on the available data from these experiments, we are unable to confidently pinpoint the mechanism through which the stiffness of the samples were affected.

There was a significant alteration in the *K*_z_ following tilting the loading direction for 15^∘^. The significantly lower stiffness of the samples in 15^∘^ abduction load cases is in line with reported lower fracture loads under similar conditions [22–24]. An interesting observation is that switching the loading configuration between neutral stance and neutral fall affects the *K*_z_ to the same extent as the 15^∘^ abduction. More importantly, this effect is comparable to that of the storage cycle. Aside from its more obvious implications in multi-directional mechanical testings, it is also noteworthy that sample misalignment of this range might significantly jeopardize the mechanical test outcomes.

Our results show that all other experimental sources of uncertainties, i.e., re-fixation, re-adjustment, pre-loading, and *μ*CT imaging had insignificant effects on the *K*_z_. In other words, replacing the samples or reassembling the testing setup are safe for the stiffness measurements. Furthermore, in the absence of standard protocols for pre-loading regimes and the possible damage they might induce in the sample, using a well-constrained setup with fully defined boundary conditions could reduce the effect of initial maladjustment between the sample and setup components, as the common reason for pre-loading cycles, and potentially alleviate the need for them. Lastly, since the popularity of *μ*CT imaging as a strong tool for hierarchical tracking of the effectiveness of in-vivo and in-vitro studies is constantly on the rise, this result could be taken as a safety indicator in terms of preservation of the structural integrity of scanned bones. It should be noted that the negligible scanning effect comes despite the 4-hour long scanning time and a moderate rise in the temperature of the chamber and sample.

Choosing the apparent sample stiffness, *K*_z_, as the main outcome variable instead of more prevalent measures of strength is adequately justifiable. Direct measurement of the bone strength, a.k.a. failure load, involves destroying the samples per test. The non-destructive alternative outcome variable to characterize a mechanical structure is its apparent stiffness (*K*) [27]. There has been shown to be a strong correlation between the stiffness and the strength of bone samples [28, 29, 35, 36]. The apparent stiffness of the bone is calculated based on the deformation of the region of interest. Strain gauges can only measure local strains, and their preparation requires substantial time and treatment of the site with possible structural damages. Full-field surface strain measurements with DIC techniques are favorable alternatives. However, with the selected maximum load threshold of 75% BW [26], at the chosen region of interest of posterior femoral neck, the noise levels proved to be high enough to prevent us from having a viable strain measurement. The same limitation resulted in limiting the number of successful marker tracking measurements and the resultant *K*_b_ values. This is in line with the reported results regarding the better performance of the DIC method in higher loading regimes and fracture tests on longer samples and at superior or inferior regions of the femoral neck, compared to < 1 BW loading cases [13, 37]. Nevertheless, there was a significant correlation between the *K*_b_ and *K*_z_ for all viable tests spanning across all load cases to merit relying on the statistical analyses of the *K*_z_ (*R*^2^ ≈ 0.92 and 0.82 for stance and fall, respectively) (Fig. 8). Given the higher sensitivity of the stiffness to structural alterations compared to the strength, which can be inferred from the lower predictive ability of models for K [14, 29, 38, 39], deducted conclusions on parameters with significant effects could even be considered as “conservative”.

There are limitations in this study that require discussion. The sample size of 10 specimens from 5 donors is relatively small. Although smaller sample sizes have been used in various studies [13, 22–24, 40], it might limit our ability to generalize the outcomes of this study to broader cases. Furthermore, pure isolation of the effects of single parameters proved to be challenging. In between the testing sets, the samples had to be taken out of the setup and put back in, resulting in the potential compound influence of the re-fixation and re-adjustment parameters in addition to those of the storage and *μ*CT scanning. However, the order-of-magnitude difference between the effect size of the storage parameter compared to those two and the similarly insignificant effect of the *μ*CT scanning leads us to deem our derived conclusions unchallenged by the interaction effect. Finally, the 5 mm/s loading rate is not representative of physiological side-fall scenarios. Although repeatedly used in relevant studies [23, 24, 41], the effect size of different studied parameters could differ under higher rates and requires further investigation.

In conclusion, the loading direction, as well as intermediary storage of the frozen samples, affect the apparent stiffness of proximal femoral samples significantly. Using a highly repeatable parametric approach, we showed that the random effects of setup manipulation and intermittent *μ*CT scanning are negligible. For multi-directional validation of FE models, a similar testing setup could be effectively used if there are no storage intervals between the different load cases on the same samples.

## Data Availability

The datasets used and/or analyzed during the current study available from the corresponding author on reasonable request.
